# Activated Notch1 Target Genes during Embryonic Cell Differentiation Depend on the Cellular Context and Include Lineage Determinants and Inhibitors

**DOI:** 10.1371/journal.pone.0011481

**Published:** 2010-07-08

**Authors:** Franziska Meier-Stiegen, Ralf Schwanbeck, Kristina Bernoth, Simone Martini, Thomas Hieronymus, David Ruau, Martin Zenke, Ursula Just

**Affiliations:** 1 Department of Biochemistry, Christian-Albrechts-University of Kiel, Kiel, Germany; 2 Department of Cell Biology, Institute for Biomedical Engineering, University Medical School, Rheinisch-Westfälische Technische Hochschule Aachen, Aachen, Germany; University of Otago, New Zealand

## Abstract

**Background:**

Notch receptor signaling controls developmental cell fates in a cell-context dependent manner. Although Notch signaling directly regulates transcription via the RBP-J/CSL DNA binding protein, little is known about the target genes that are directly activated by Notch in the respective tissues.

**Methodology/Principal Findings:**

To analyze how Notch signaling mediates its context dependent function(s), we utilized a Tamoxifen-inducible system to activate Notch1 in murine embryonic stem cells at different stages of mesodermal differentiation and performed global transcriptional analyses. We find that the majority of genes regulated by Notch1 are unique for the cell type and vary widely dependent on other signals. We further show that Notch1 signaling regulates expression of genes playing key roles in cell differentiation, cell cycle control and apoptosis in a context dependent manner. In addition to the known Notch1 targets of the Hes and Hey families of transcriptional repressors, Notch1 activates the expression of regulatory transcription factors such as Sox9, Pax6, Runx1, Myf5 and Id proteins that are critically involved in lineage decisions in the absence of protein synthesis.

**Conclusion/Significance:**

We suggest that Notch signaling determines lineage decisions and expansion of stem cells by directly activating both key lineage specific transcription factors and their repressors (Id and Hes/Hey proteins) and propose a model by which Notch signaling regulates cell fate commitment and self renewal in dependence of the intrinsic and extrinsic cellular context.

## Introduction

The Notch signaling pathway is a highly conserved signaling mechanism that controls cell fate decisions, proliferation and apoptosis during development and in the adult [1], [2]. In mammals, Notch proteins comprise a family of four transmembrane receptors (Notch1-4). Specific transmembrane ligands (Jagged-1, Jagged-2, Delta-like-1, Delta-like-3, and Delta-like-4) interact with Notch receptors on neighboring cells. Activating ligands induce cleavage near the transmembrane region of the Notch intracellular domain (Notch^IC^) resulting in the release and nuclear translocation of Notch^IC^ [1]. Nuclear Notch^IC^ interacts with the transcriptional repressor RBP-Jκ (RBP-J/CSL/CBF1/Su(H)/Lag1), and converts it into an activator [3], leading to the expression of direct Notch target genes [4].

The outcome of Notch signaling is highly dependent on the cellular context [1]. Notch activity affects differentiation, proliferation, and apoptotic programs in concert with other cell-intrinsic or cell-extrinsic developmental cues that are necessary to execute specific developmental programs [1]. However, despite the identification of many interacting pathways [4], it remains unclear how the highly variable, context-specific effects of Notch signaling are integrated at the molecular level, i.e. which specific target gene programs are activated.

The best characterized direct targets of Notch signaling are the Hes (Hairy/Enhancer of Split) and Hey (also called Herp/Hesr/Hrt/CHF/gridlock) families of basic helix-loop-helix (bHLH)-type transcriptional repressors [5], [6]. Notch/RBP-J signaling activates Hes/Hey transcription, which leads to repression of Hes/Hey target genes such as tissue-specific transcriptional activators, thereby preventing differentiation [5]. More recently, several other genes with quite diverse functions have been found to be directly regulated by Notch signaling [7], [8], implying that Notch exerts its pleiotropic functions by acting through multiple specific targets.

Early mammalian development is characterized by a series of events resulting in the formation of the three germ layers, ectoderm, mesoderm and endoderm, which later segregate and further differentiate to form mature tissues. Components of the Notch pathway are present in mammalian cells during the early stages of embryogenesis [9], [10] and correct Notch signals are required for normal early embryonic development [11]–[13]. We and others have shown that Notch blocks mesodermal differentiation at the initial stages of embryonic stem cell (ESC) differentiation and promotes neuroectodermal commitment when these cells are cultured in the absence of self renewal and serum factors, suggesting that Notch signaling plays a role during the specification of the germ layers during mammalian embryogenesis [10], [14], [15]. At a later stage during mesodermal differentiation, in Flk1 receptor expressing mesodermal progenitor cells, Notch signaling inhibits the generation of muscle, endothelial and hematopoietic cells and favors the generation of mural cells [14].

To examine the cell context-dependent regulation of Notch target genes systematically, we have performed genome-wide transcriptome analyses of Notch1-induced genes in murine ESC under different cell extrinsic cues and in mesodermal cells. We show that Notch signaling activates expression of genes involved as key factors in cell differentiation, cell cycle control and apoptosis in a highly cell-extrinsic and cell-intrinsic cell-context dependent manner. In addition to the classical immediate Notch downstream genes of the Hes and Hey famliy of transcriptional repressors, we identified several key transcription factors such as Sox9, Pax6, Runx1, Myf5 and Id (inhibitor of DNA binding or differentiation) proteins that are critically involved in lineage decisions as differentially regulated Notch1 target genes. Based on our findings we propose a model for a cell-context dependent regulatory network controlling cell fate that involves integration of Notch and other cell-extrinsic and cell-intrinsic signals to fine-tune the level of expression of positive and negative lineage determinants for timed, cell-context dependent lineage-decisions.

## Results and Discussion

### Activation of Notch1^IC^ induces expression of specific target genes in embryonic stem cells and mesodermal cells in a highly cell-intrinsic and cell-extrinsic context dependent manner

**Figure 1:**
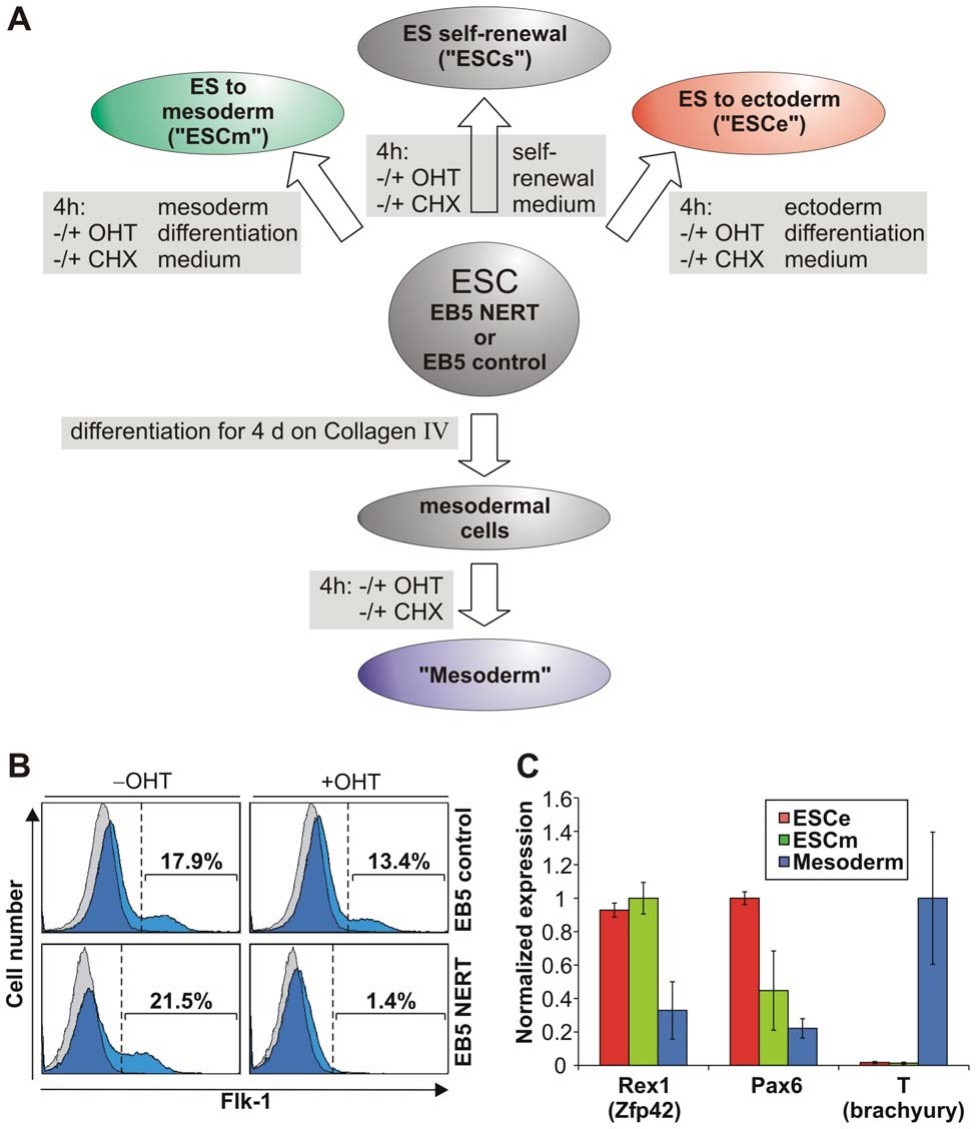
Cell populations used for investigation of Notch1 signaling. (A) Scheme of cell populations and culture conditions for identification of Notch1 target genes. Undifferentiated EB5 NERT or EB5 control ESC were kept under self-renewal conditions (ESCs) and treated with OHT for 4 h. Alternatively, ESC were cultured in ectodermal differentiation medium (ES to ectoderm; ESCe) or mesodermal differentiation medium (ES to mesoderm; ESCm) during OHT treatment. Furthermore, ESC were differentiated for 4 d on Collagen IV to mesodermal cells (Mesoderm), which were then treated by OHT for 4 h. To distinguish direct from indirect targets, all experiments were additionally performed with or without CHX to inhibit protein synthesis. After 4 h OHT treatment, RNA was extracted from the different cell populations and subjected to further analysis. (B) Mesodermal differentiation of ESC and its suppression by activated Notch1. EB5 control or EB5 NERT cells were cultured for 4.5 d in mesodermal differentiation medium on OP9 stromal cells with or without OHT and the percentage of Flk1+ cells was analyzed by flow cytometry. Histograms of the cells stained with anti-Flk-1 antibody are depicted in blue and histograms of the cells stained with isotype matched mouse IgG are depicted in grey. The area left to the dotted line indicates Flk-1+ cells. The percentage of Flk-1+ cells is shown for each histogram. One representative example of eleven (EB5 NERT cells) or four (EB5 controls cells) experiments is shown, respectively. Reduction of Flk+ positive cells is statistically significant (p<0.01). (C) Expression of Rex1, Pax6, and T (brachyury), which characterize undifferentiated ESC, ectodermal cells and mesodermal cells, respectively, in the cell populations used for array analyses. The different cell types described in A were evaluated for RNA expression of key pluripotency and differentiation genes via microarray analysis. The expression was normalized to the highest mean. Error bars indicate standard deviation. Since Pax6 was identified as a Notch1 target, OHT induced samples were not included for calculation of this gene.

To identify cell-context dependent Notch target genes during ESC differentiation, we employed a conditional Notch activation system that allows the timed activation of Notch1 signaling by the addition of 4-hydroxy-tamoxifen (OHT) in an *in vitro* developmental model system closely resembling *in vivo* development [14]. Using EB5 ESC carrying an OHT-inducible form of murine Notch1^IC^ (EB5 NERT [14]) or control cell lines carrying the pCAG expression vector without the NERT cDNA (EB5 control), the genome-wide, activated Notch1 induced changes in the expression of genes in ESC cultured in differentiation-inducing conditions favoring either mesodermal (ESCm) or ectodermal (ESCe) differentiation or in mesodermal cells (Mesoderm) were determined (Fig. 1A). Based on earlier studies on the induction of previously identified Notch1 target genes and on the time required for activated Notch1 to induce cell lineages decisions in our OHT-dependent systems [14], [16]–[18], we chose a 4 h induction period with OHT. To further test whether the identified genes are likely direct targets of activated Notch1, we determined mRNAs regulated by Notch1 signaling in the absence of *de novo* protein synthesis. The efficient mesodermal differentiation and biological activity of Notch1 activation at the time of RNA isolation was confirmed by differentiating the cells on either OP9 cells or collagen IV and activating Notch1 signaling by OHT (Fig. 1B). In line with our recent study [14], activation of Notch1 signaling by the addition of OHT strongly reduced the generation of Flk1^+^ mesodermal progenitor cells from EB5 NERT ESC (Fig. 1B), while OHT treatment had no effect on the differentiation of EB5 control ESC (Fig. 1B). Pluripotency of ESC and correct differentiation were further confirmed by monitoring the expression of *Rex1* (*Zfp42*), a gene commonly used as a landmark of pluripotency, and the expression of genes indicative of ectoderm, i.e. *Pax6*, or of mesoderm, i.e. Brachyury (*T*), respectively (Fig. 1C).

**Figure 2:**
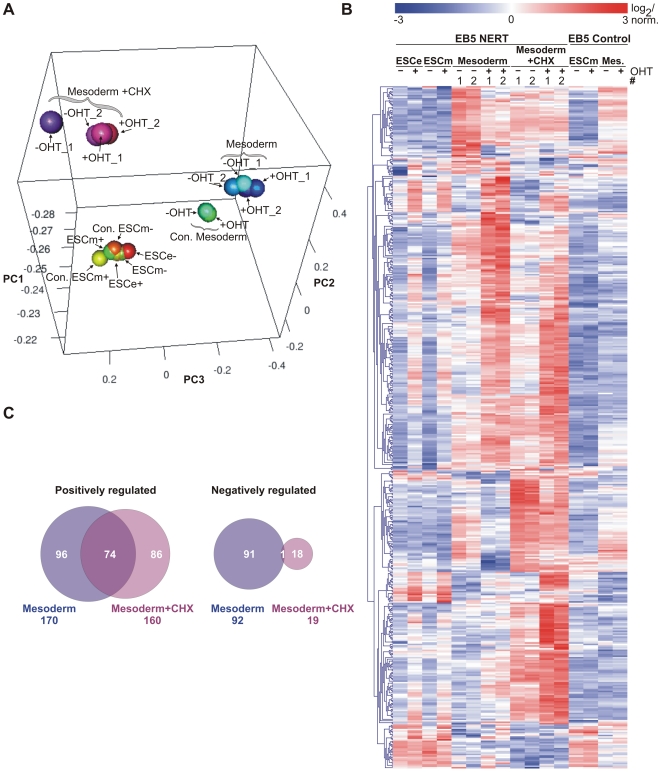
Identification of cell-context specific Notch1 target genes by genome wide gene expression arrays. (A) 3-dimensional principle component analysis (3D PCA). Notch1 signaling was induced with OHT for 4 h in EB5 NERT or EB5 control cells (Con.) as outlined in Figure 1A. Mesodermal cells (Mesoderm) were additionally treated with or without CHX. The various culture conditions clearly cluster to different areas in the plot, whereas differences induced by Notch1 are rather subtle. An animated presentation of the 3D PCA is available in the supplemental material. (B) Expression heatmap of the Notch1 induced genes. 465 transcripts were identified as differentially expressed due to Notch1 induction in at least one of the conditions (ESCe, ESCm, Mesoderm (Mes.), or Mesoderm+CHX) according to the criteria outlined in Material and Methods. Expression values of the samples were Log_2_ transformed and normalized within each gene. (C) Venn diagram of Notch1 induced genes in mesodermal cells in the presence or absence of CHX. Of the 262 genes regulated without CHX treatment, 170 are up-regulated and 92 are down-regulated, whereas with CHX treatment 160 of the 179 differentially expressed genes were positively and 19 were negatively regulated. Importantly, there is a significant overlap of 74 up-regulated genes indicating potential direct target genes of Notch1 in mesodermal cells, whereas there is almost no overlap for the down-regulated genes. Thus, positive regulation seems to be the main mechanism of Notch1 gene regulation, whereas the negative regulation observed accounts for indirect effects.

First, we analyzed the gene array data by principal component analysis (PCA) that clusters data sets according to their degree of correlation (Fig. 2A and Video S1). PCA shows that samples of each cell population, i.e. ESC (ESCe and ESCm) and mesodermal cells (Mesoderm), cluster together, regardless of the expression of the NERT protein or the presence of OHT. While control (Con.) and NERT expressing ESC cluster closely together, mesodermal cells cluster slightly apart in control (Con.) and NERT expressing cells, probably because kinetics of mesodermal differentiation varies in individual clones. Cells treated with cycloheximide (CHX) cluster separately from both ESC and mesodermal cells, indicating that blocking protein synthesis profoundly alters the gene expression profile. Importantly, OHT did not influence clustering in any of the cell populations, suggesting that neither the addition of OHT nor Notch signaling globally influences gene expression.

Next we investigated the changes in gene expression by the induction of Notch1 signaling. Based on a signal log ratio of 1 (at least 2-fold change in gene expression), 401 annotated genes and 64 expressed sequences were regulated in EB5 NERT cells by the addition of OHT (Fig. 2B and Table S1). None of these genes was regulated by OHT in EB5 control cells (Figs. 2B and Table S1), suggesting that these genes are bona fide Notch target genes. In line with its signal transduction mechanism as a transcriptional activator, activated Notch1 mostly up-regulated, rather than down-regulated, the expression of target genes (Fig. 2C).

**Figure 3:**
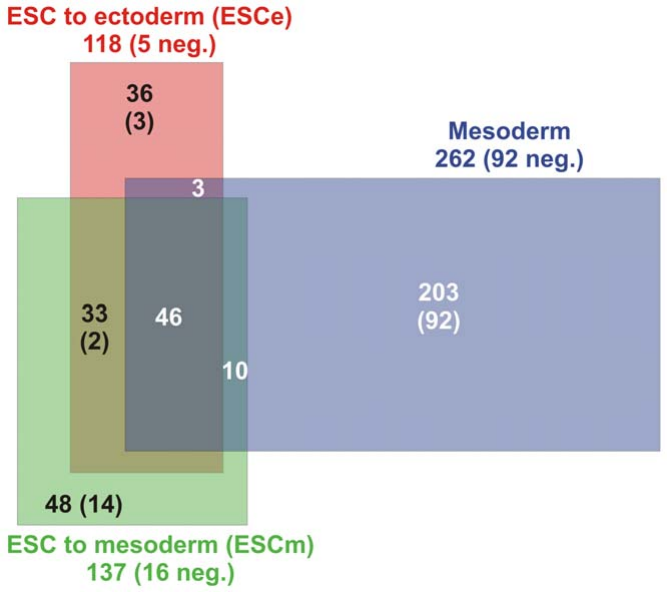
Cell-context specific Notch1 target genes identified by genome wide gene expression arrays. Venn diagram of differentially Notch1 induced genes in ESC to ectoderm (ESCe), ESC to mesoderm (ESCm), or mesodermal cells (Mesoderm). Numbers at the labeling indicate the total number of regulated genes. Numbers in the areas indicate the overlap of the different conditions. All numbers in parentheses show the quantity of negatively regulated genes.

Importantly, the majority of Notch1 regulated genes were unique for the cell type, i.e. for ESC (ESCe and ESCm) and mesodermal cells, respectively, and about half of the Notch1 regulated genes in ESC were specific for the culture condition used, i.e. media favoring either mesodermal or ectodermal differentiation (Fig. 3). Only about 10% of all identified Notch1 regulated genes were common for the different cell populations and culture conditions analyzed (Fig. 3). These results demonstrate that Notch/RBP-J signaling regulates different sets of genes in different cell types. Furthermore, other signaling pathways induced by the culture supplements strongly influence which target genes are activated by Notch/RBP-J signaling.

To validate further our target gene identification, we compared the Notch1 target genes identified in ESC cultured in media favoring ectodermal differentiation (ESCe) with the Notch1 target genes identified recently in another study in ESC cultured under similar culture conditions [19]. About 44% of the activated genes in our ESCe analysis were also activated in the study by Main et al [19], thus revealing a considerable overlap of Notch1 targets in the same cell type and under similar differentiation conditions despite the use of different ESC (EB5 vs. 46C), different Notch1 induction systems (induction of nuclear translocation of the Notch^IC^-ERT fusion protein by OHT vs. induction of Notch^IC^ expression by the Tet system) and different time point of analyses (4 h induction vs. 6 h induction). The high overlap of Notch1 target genes identified in similar cell type and culture conditions in two separate studies with different approaches of Notch activation together with the high cell type and context dependency of Notch1 target genes identified in the present study suggests that Notch target gene induction is tightly regulated, despite its cell-context dependency.

**Figure 4:**
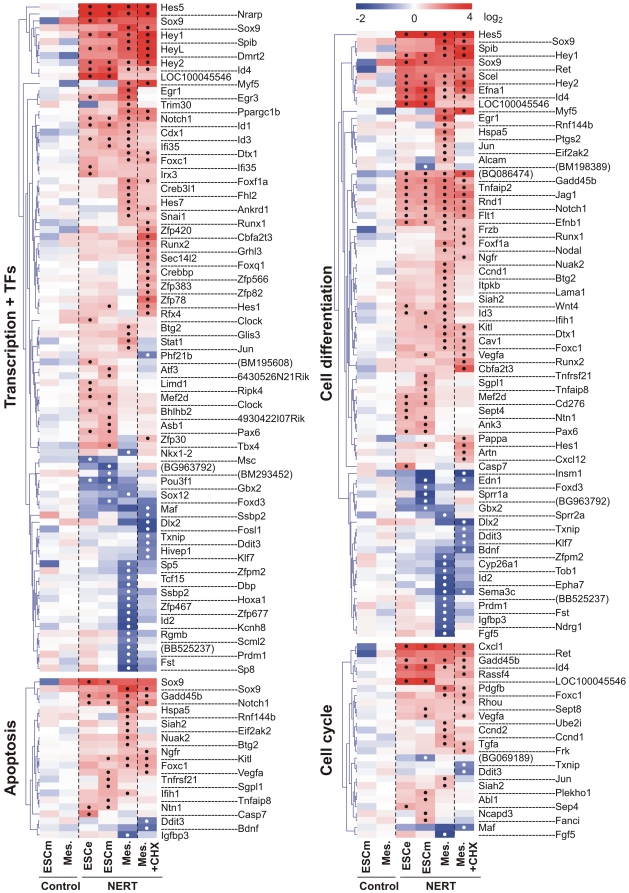
Induction heat maps of Notch1 target genes involved in transcription, apoptosis, cell cycle and cell differentiation. The identified Notch1 targets were grouped into different Gene Ontology (GO) terms using the DAVID functional annotation analysis tool (http://david.abcc.ncifcrf.gov/). Note that only differentially regulated genes by Notch1 are shown as Log_2_ transformed values. If no gene symbol was available for the regulated transcript, GenBank accession number was given in parentheses. Significant changes of differentially regulated gene expression according to the criteria outlined in Material and Methods were labeled with a black or white dot for up- and down-regulated genes, respectively. Redundant probe sets for the same gene were removed except when significantly different results were obtained (e.g. Sox9). Pcdha@*) indicates the protocadherin αcluster comprising *Pcdha1, Pcdha2, Pcdha3, Pcdha4, Pcdha5, Pcdha6, Pcdha7, Pcdha8, Pcdha9*, *Pcdha10, Pcdha11, Pcdha12, Pcdhac1, Pcdhac2, and Pcdhgb2*. The complete list of regulated genes is available in the Supplement Table S1.

**Figure 5:**
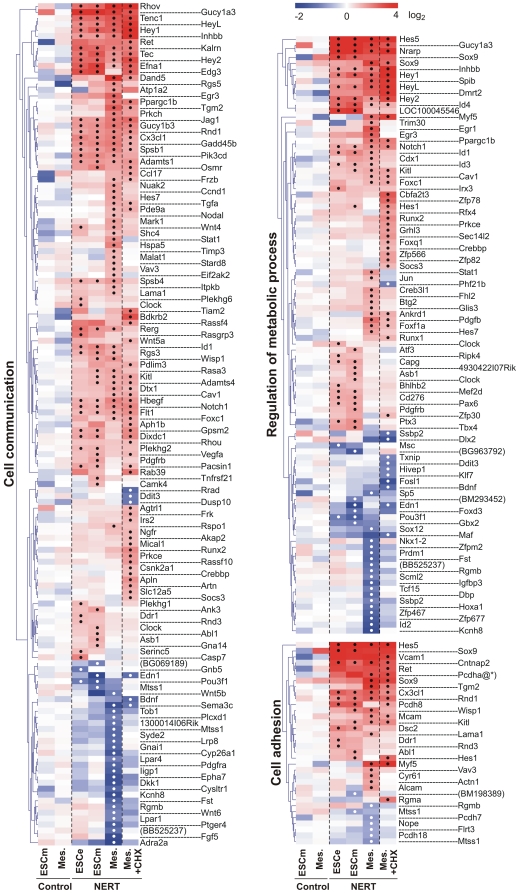
Induction heat maps of Notch1 target genes involved in regulation of metabolic process, cell adhesion and cell communication. The identified Notch1 targets were grouped into different Gene Ontology (GO) terms using the DAVID functional annotation analysis tool (http://david.abcc.ncifcrf.gov/). Note that only differentially regulated genes by Notch1 are shown as Log_2_ transformed values. If no gene symbol was available for the regulated transcript, GenBank accession number was given in parentheses. Significant changes of differentially regulated gene expression according to the criteria outlined in Material and Methods were labeled with a black or white dot for up- and down-regulated genes, respectively. Redundant probe sets for the same gene were removed except when significantly different results were obtained (e.g. Sox9). The complete list of regulated genes is available in the Supplement Table S1.

**Figure 6:**
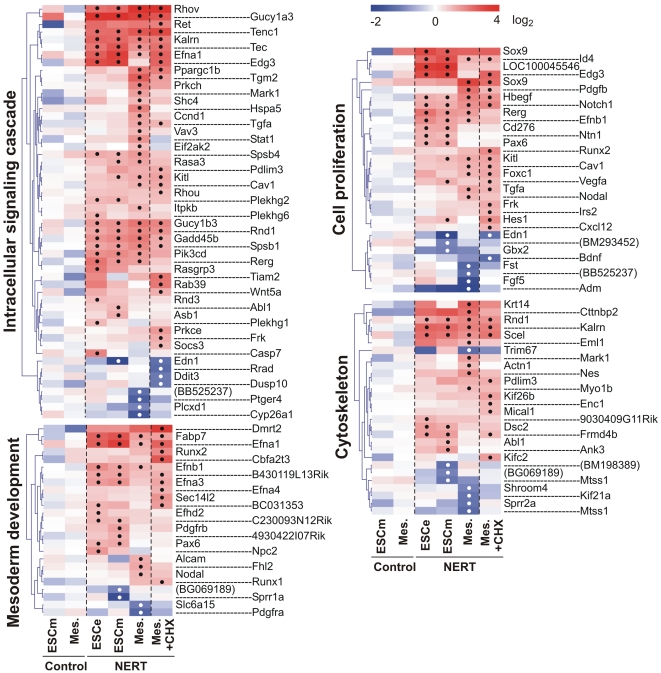
Induction heat maps of Notch1 target genes involved in mesoderm development, intracellular signalling cascade, cell proliferation and cytoskeleton. The identified Notch1 targets were grouped into different Gene Ontology (GO) terms using the DAVID functional annotation analysis tool (http://david.abcc.ncifcrf.gov/). Note that only differentially regulated genes by Notch1 are shown as Log_2_ transformed values. If no gene symbol was available for the regulated transcript, GenBank accession number was given in parentheses. Significant changes of differentially regulated gene expression according to the criteria outlined in Material and Methods were labeled with a black or white dot for up- and down-regulated genes, respectively. Redundant probe sets for the same gene were removed except when significantly different results were obtained (e.g. Sox9). The complete list of regulated genes is available in the Supplement Table S1.

**Figure 7:**
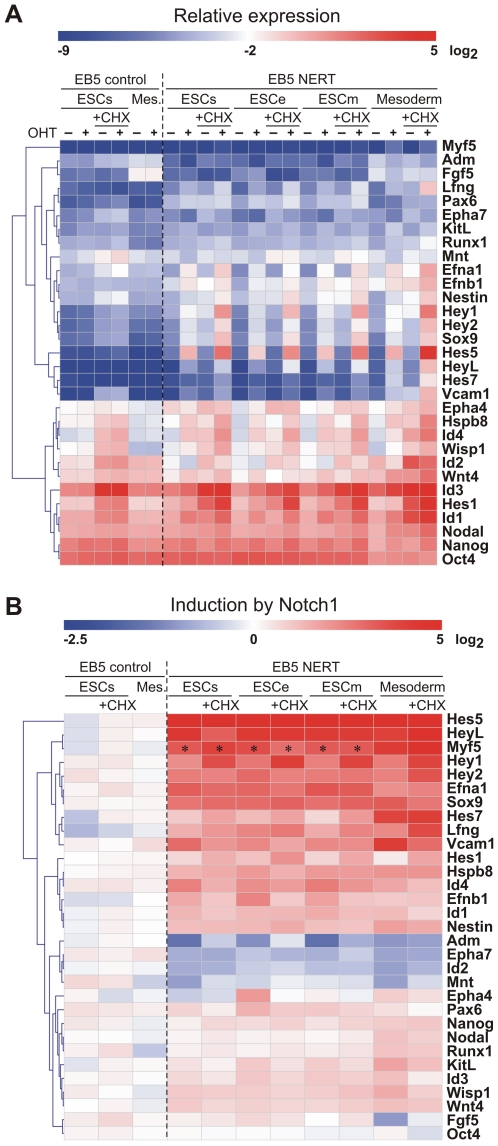
Notch1 target genes validated by qPCR. The microarray results were validated by qPCR using ESCs, ESCe, ESCm and Mesoderm. All conditions were tested also in the absence or presence of cycloheximide (+CHX). *Oct4* (last gene; also known as *Pou5f1*) is included as negative non-regulated control. (A) Mean values of relative expression (% of *Gapdh* expression, Log_2_ transformed) shown as a heatmap in the absence (−) or presence (+) of OHT. (B) Inductions by activated Notch1 were calculated from relative expression values and were Log_2_ transformed. Thus, blue areas indicate down-regulation and red regions show up-regulation. *) indicates low level expression of *Myf5* near detection limit resulting in artificially high induction values that are not reliable.

Genes targeted by activated Notch1 encompassed a wide variety of intracellular and extracellular regulatory proteins, including cell lineage determinants, transcription factors, cell cycle regulators, intracellular signaling mediators and receptors and ligands, reflecting the pleiotropic functions of Notch (Fig. 4, 5, 6). In line with the short time of induction, most genes were regulated by activated Notch1 in the presence of CHX as well and thus present likely direct target genes of Notch1 (Fig. 4, 5, 6). To confirm our gene expression array data, 30 genes that were differentially regulated by Notch1 signaling in our array analyses were examined by qPCR. Gene expression values of EB5 NERT and EB5 control cells under differentiating (ESCe and ESCm) and self-renewing conditions (ESCs) and in mesodermal cells (Mesoderm) grown in the presence or absence of OHT and in the presence or absence of CHX for 4 h were obtained (Fig. 7). In line with our array data, none of the genes analyzed was regulated by OHT in EB5 control cells and all showed a comparable pattern of regulation in EB5 NERT cells following the addition of OHT (Fig. 4, 5, 6, 7, Table S1). As expected, *Hes* and *Hey* genes showed a similar or even higher induction of mRNA expression by activated Notch1 in the presence of CHX (Fig. 4, 5, 6, 7, Table S1), confirming that *Hes* and *Hey* genes are direct targets of Notch signaling. Interestingly, the well known Notch target genes *Hes1* and *Hes7* were also cell specifically induced by activated Notch1: In the absence of CHX, the expression of *Hes1* was only up-regulated by activated Notch1 in ESC but not in mesodermal cells, while *Hes7* was up-regulated only in mesodermal cells but not in ESC (Fig. 4, 5, 6, 7 and Table S1). Taken together, we conclude that Notch1 exerts its multiple effects by specifically regulating the expression of genes in a highly cell intrinsic and cell extrinsic context dependent manner.

### Notch1/RBP-J signaling induces expression of key transcriptional regulators involved in cell lineage determination in the absence of protein synthesis

**Figure 8:**
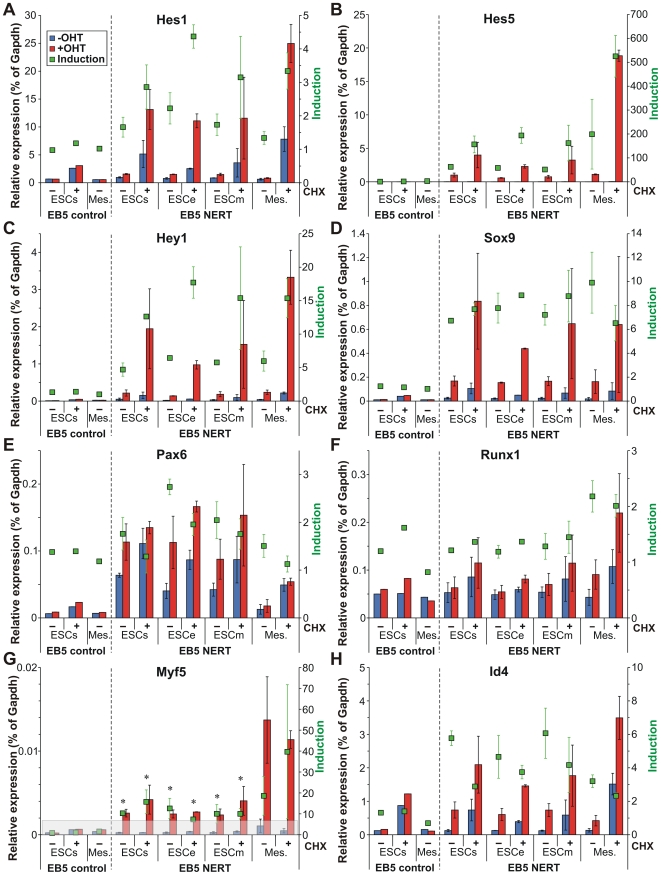
Regulatory transcriptions factors involved in differentiation are induced by activated Notch1 in the absence of protein synthesis. (A–H) The microarray results were validated by qPCR in ESC cultured in self renewal (ESCs), ectodermal (ESCe), or mesodermal (ESCm) culture medium and in mesodermal cells (Mes.). All conditions were tested in the absence (−) or presence (+) of CHX. Relative expression is shown for untreated (*blue bars*) and OHT treated samples (*red bars*) compared to *Gapdh*. The calculated inductions are indicated by *green squares*. The error bars indicate the standard deviation from 2 to 6 independent experiments for the cells carrying the inducible Notch1 (NERT). *Myf5* (G) is expressed at a very low level in undifferentiated cells (*ESCs*, *ESCe*, *ESCm*). Calculated inductions that do not reach significance above background levels (*grey area*) are indicated by an asterisk.

**Figure 9:**
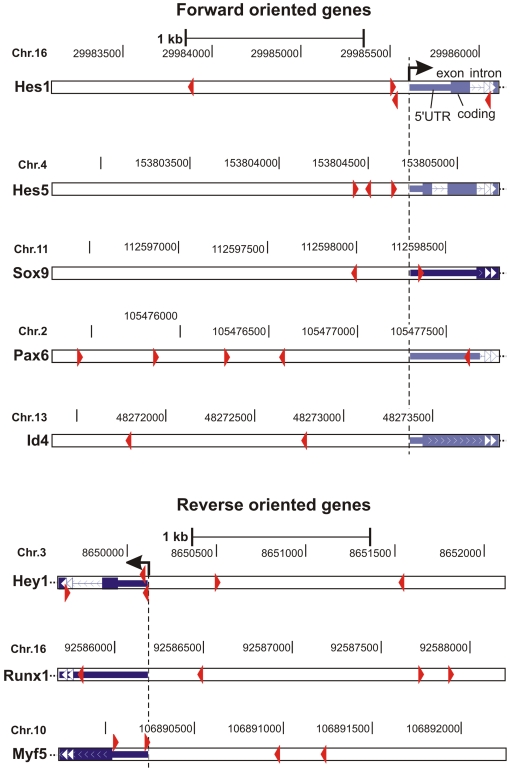
RBP-J binding site sequences of potential direct Notch1 target genes. Regulatory regions comprising 2000 bp before to 500 bp after the transcription start site of the Notch1 target genes Hes1, Hes5, Sox9, Pax6, Id4, Hey1, Runx1, and Myf5 were screened for potential RBP-J binding sites (red arrow head indicates direction of binding) using the ‘Transcription Element Search System’ (TESS; http://www.cbil.upenn.edu/tess; [67]) and a weight matrix from Ong et al. [68]. As a threshold, a Lg-likelyhood score (La) of 10 had to be exceeded. Binding sites were projected onto respective genomic regions using the UCSC Genome Browser (http://genome.ucsc.edu/) using the Feb. 2006 (NCBI36/mm8) assembly.

The major function of Notch signaling is to regulate differentiation decisions. Classically, this pathway is employed to restrict cell differentiation by signaling through the Hes/Hey transcriptional repressors. In this study however, we found that a number of further key regulatory transcription factors essential for lineage determination and cell differentiation were induced by Notch1 signaling in a cell context dependent manner (Figs. 4, 6, 7). These included transcription factors that play a role in all three germ layers, such as Sox9, transcription factors that are essential specifically in neuro-ectodermal development, such as Pax6, and transcription factors required for lineage specification of mesodermal derived tissues, such as Myf5 for skeletal muscle development and Runx1 for hematopoietic stem cell fate of mesodermal progenitor cells. Furthermore, Notch1/RBP-J signaling differentially regulated expression of members of the Id family of transcriptional repressors that interfere with the transcriptional activities of differentiation inducing transcription factors. Importantly, all of these genes were also induced by activated Notch1 to a similar or even higher extent in the absence of protein synthesis (Fig. 7, 8), suggesting that they represent direct target genes of Notch1. To further analyze a potential direct regulation, the promoter sequences of these genes were screened for RBP-J binding site sequences. As shown in Figure 9, the promoters of these transcription factors contained consensus RBP-J binding sites, thus supporting a model by which Notch signaling influences lineage decisions by directly activating key transcriptional regulators involved in cell lineage determination (see model below).

### The transcription factor Sox9 is a primary Notch1 target gene

**Figure 10:**
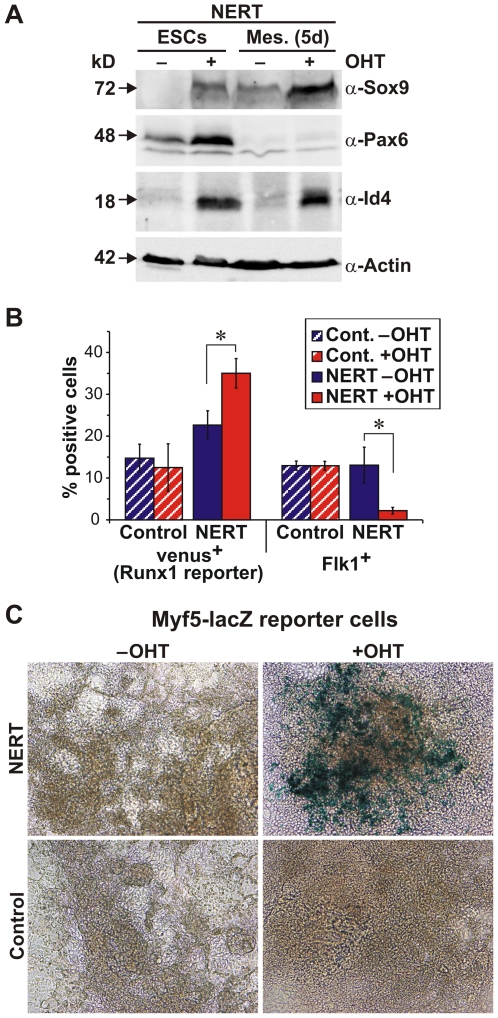
Validating the Notch1 target genes Sox9, Pax6, Id4, Runx1 and Myf5. Notch1 signaling was induced in ESC and differentiating embryoid bodies by addition of OHT (500 nM). (A) Immunoblot of EB5 NERT ESC (NERT ESCs) and EB5 NERT differentiated embryoid bodies (NERT Mes. 5d) with Sox9, Pax6 and Id4 antibodies. One representative example of three independent experiments is shown. (B) Mesodermal differentiation of Runx1-Venus-NERT ESC or Runx1-Venus control ESC and analysis of the percentage of venus^+^ and Flk1^+^ cells by FACS. Error bars indicate standard deviation for three independent experiments. (C) Mesodermal differentiation of Myf5-lacZ-NERT or Myf5-lacZ control ESC and detection of LacZ expression. One representative example of three independent experiments on day 5 is shown. Virtually identical results were obtained when the cells were analyzed 4 or 7 days, respectively, after induction of differentiation. Addition of OHT at day 3 of embryoid body culture similarly yielded in an increased number of lacZ positive cells at days 4, 5 and 7 in the presence of OHT after induction of differentiation.

Among the genes that were induced by activated Notch1 in undifferentiated ESC (ESCs), in ESC differentiating along the ectodermal (ESCe) or mesodermal (ESCm) lineages as well as in mesodermal cells (Mesoderm) was *Sox9* (Fig. 4, 7, 8D), an essential transcription factor involved in development of all three germ layers. In line with our RNA data, activated Notch1 shifted Sox9 protein expression from undetectable to an intermediate level in ESCs (Fig. 10A) and in 5 d differentiated embryoid bodies from an intermediate to high level expression (Fig. 10A), confirming a valid role of Notch for Sox9 regulation in both undifferentiated and differentiated cells. Interestingly, RBP-J has been shown to be required to maintain *Sox9* expression during the gliogenic phase of spinal cord development [20] and Notch signaling is essential for Sox9 expression in the mouse retina [21], however whether Notch signaling directly or indirectly regulates *Sox9* expression has not been determined. Here we show that Sox9 is upregulated by activated Notch1 even when protein synthesis is inhibited by CHX (Fig. 4, 7, 8D). Hence, Sox9 appears to be a direct target of Notch1. Recently, Notch has been shown to bind to the Sox9 proximal promoter in liver cells [22], further supporting a direct link between Sox9 and Notch1. Furthermore, suppression of the Notch1 induced Sox9 upregulation using specific siRNA reverted Notch1 induction of chrondogenesis (S.M., R.S., U.J., R. Haller, J. Kramer, J. Rohwedel, manuscript in preparation), demonstrating Sox9 as critical mediator of Notch-induced cell lineage decisions. Taken together, the direct regulation of Sox9 by activated Notch1 may play an important role for the development of cells in all three germ layers.

### The neuro-ectodermal transcription factor Pax6 is induced by Notch1 signaling in ESC but not in mesodermal cells

Recently, we and others have shown that the activation of Notch has a decisive role in ES cell fate determination [14], [15]: Notch suppresses the generation of mesodermal cells from ES cells and promotes neural commitment of ES cells, when cultured in the absence of self renewal and serum factors, suggesting a role for Notch during the specification of the germ layers during mammalian embryogenesis. How Notch mediates this cell lineage specification at the molecular level is currently unknown. Here we have identified the neuroectodermal transcriptional regulator *Pax6* as a Notch1 target gene in ESC but not in mesodermal cells (Fig. 4, 7, 8E). Moreover, the observed up-regulation of Pax6 by activated Notch1 was most likely direct in that the up-regulation was not affected by CHX and, thus, independent of protein synthesis (Fig. 4, 7, 8E). The effect of cell-context dependent Notch regulation was further paralleled by Pax6 protein levels (Fig. 10A). Pax6 is crucial for various developmental processes in the central nervous system and other ectodermal tissues [23]. Depending on the cellular context it promotes cell proliferation and expansion of neural stem cells, or neuronal differentiation. Importantly, expression of Pax6 in ESC favors neuroectodermal lineage choice and radial glia formation [24], in a similar way to activated Notch1. Furthermore, in *Drosophila* and *Xenopus*, Notch signaling also induces eye-related gene expression, including *Pax6* [25]. Taken together, it is thus conceivable that the direct activation of *Pax6* expression is involved in Notch-induced differentiation of ESC into radial glia in a context-dependent manner.

### The mesodermal transcription factor Runx1 is induced by Notch1 signaling in mesodermal cells but not in ESC

*Runx* genes are key regulators of lineage-specific gene expression in major developmental pathways. The expression of *Runx* genes is tightly regulated, leading to distinct tissue- and developmental-specific expression patterns [26]. Here we identified *Runx1* (AML1) as a Notch1 target gene in mesodermal cells but not in ESC (Fig. 4, 7, 8F). Because Runx1 was induced by activated Notch1 in the presence of CHX, i.e. without protein synthesis, Runx1 is likely to be a direct target gene of Notch (Fig. 4, 7, 8F). Runx1 determines commitment of mesodermal progenitor cells to the hematopoietic lineage [27]. In line with our findings, studies in *Drosophila*, zebrafish and mouse have defined a Notch-Runx1 pathway to be critical for developmental specification of hematopoietic stem cell fate and homeostasis of hematopoietic stem cell number [28]–[30]. However, although these studies clearly established the dependency of Notch signaling on *Runx1* up-regulation for hematopoietic stem cell fate and maintenance, neither study showed direct transcriptional regulation of *Runx1* by activated Notch1. Taken together, we propose that activated Notch influences mesodermal progenitor cells to adopt a hematopoietic stem cell fate by a mechanism involving the cell-context dependent transcriptional up-regulation of *Runx1* gene expression.

### The myogenic transcription factor Myf5 is induced by Notch1 signaling in mesodermal cells but not in ESC

Notch signaling plays an important role in the maintenance of muscle progenitor cells during embryogenesis and in the generation and maintenance of satellite cells, the stem cells of mature muscle, in fetal development as well as during regeneration in the adult [31]. The myogenic program is controlled by the combinatorial activity of myogenic regulatory factors comprising Myf5, Myod1, Myogenin and MRF4/Myf6/Herculin which, together with paired domain transcription factors Pax3 and Pax7, are essential for skeletal myogenesis [32]. In *RBP-J* or *Delta1* deficient mice, muscle progenitor cells show premature differentiation, leading to depletion of the progenitor pool [33], [34]. Since it has been shown that Delta/Notch/RBP-J signaling represses the expression of the *Myod1* via activation of Hes1, thereby blocking muscle differentiation [35], and that *Myod1* deficiency leads to increased survival of muscle stem cells [36], one way by which Notch signaling maintains the muscle stem cell pool may be by the down-regulation of Myod1. In our screen for Notch target genes, however, we have identified *Myf5* as a potential direct Notch1 target gene in mesodermal cells (Fig. 4, 7, 8G). Myf5 is expressed first in the paraxial mesoderm and later in skeletal muscle progenitor cells, and is followed by the expression of Myod1. Myf5 and Myod1 determine two genetically and lineally distinct populations of muscle progenitor cells, which can substitute for each other within the developing embryo [37], [38]. Myf5 supports efficient skeletal muscle regeneration by promoting satellite cell proliferation [39] and may be involved in the expansion of the progenitor cell pool. Considering the roles of Myf5 and Myod1 for muscle development, we propose that the direct induction of *Myf5* expression together with Hes1-mediated down-regulation of *Myod1* by Notch signaling may contribute to the maintenance of muscle progenitor cells. Along this line, the up-regulation of *Myf5* and down-regulation of *Myod1* by ligand-activated Notch signaling has been demonstrated in C2C12 myoblasts [40]. Thus, there may be several cooperating mechanisms by which Notch signaling maintains the muscle progenitor pool during embryogenesis and in the adult.

### Transcriptional repressors of the Id family are differentially regulated by Notch1 signaling in a cell-context dependent manner

In addition to lineage specific transcription factors we found members of the Id family of bHLH transcriptional repressors to be induced by Notch1 signaling (Fig. 4, 7, 8H, 10A). Induction of Id genes was significant also in the presence of CHX (Fig. 4, 7, 8H). Thus, like the lineage specific transcription factors, the Id genes are likely direct Notch1 targets. Id proteins function as dominant negative regulators of other bHLH, Ets or Pax transcription factors, which positively regulate differentiation in many cell lineages [41]. Id proteins, which lack a DNA-binding domain, heterodimerize with other transcription factors, either the ubiquitous E proteins or HLH activators, thereby antagonizing their function. In *Drosophila*, a strong genetic interaction has been found between *emc* genes, the Drosophila homologue for the Id proteins, and genes that encode different components of the Notch signaling pathway [42]. Along this line, Notch signaling has been shown to positively regulate the transcription of XId3, a *Xenopus* member of Id proteins, through a Su(H)-dependent pathway [43]. In this study we found several members of the Id family to be differentially regulated by activated Notch1: While *Id1*, *Id3* and *Id4* were up-regulated, *Id2* was down-regulated by activated Notch1 (Fig. 4, 7). The significance of this differential regulation is not clear. Id1, Id2 and Id3 show a similar expression pattern during embryogenesis, while the distribution of Id4 is distinct [44]. Due to functional redundancy among the four members of the Id family and their widespread, overlapping expression patterns, only crosses between mice that lack different *Id* genes to generate multiple knock-outs are embryonic lethal. Id factors have been implicated to play an essential role mostly during early development, in neural development, hematopoiesis and angiogenesis. With regard to Notch signaling, it is of interest to note that Id proteins are short-lived proteins that have been reported to function as intracellular timer of differentiation in the nervous system [44]. It is thus conceivable that Notch upregulates certain Id proteins, in particular Id4 (Fig. 10A), a crucial regulator of neural stem cell fate determination, to control timing of differentiation (see model below).

### Genetic complementation assays and knock-in reporter cell lines confirm Notch1 target genes identified in the Notch1-induced transcriptome

**Figure 11:**
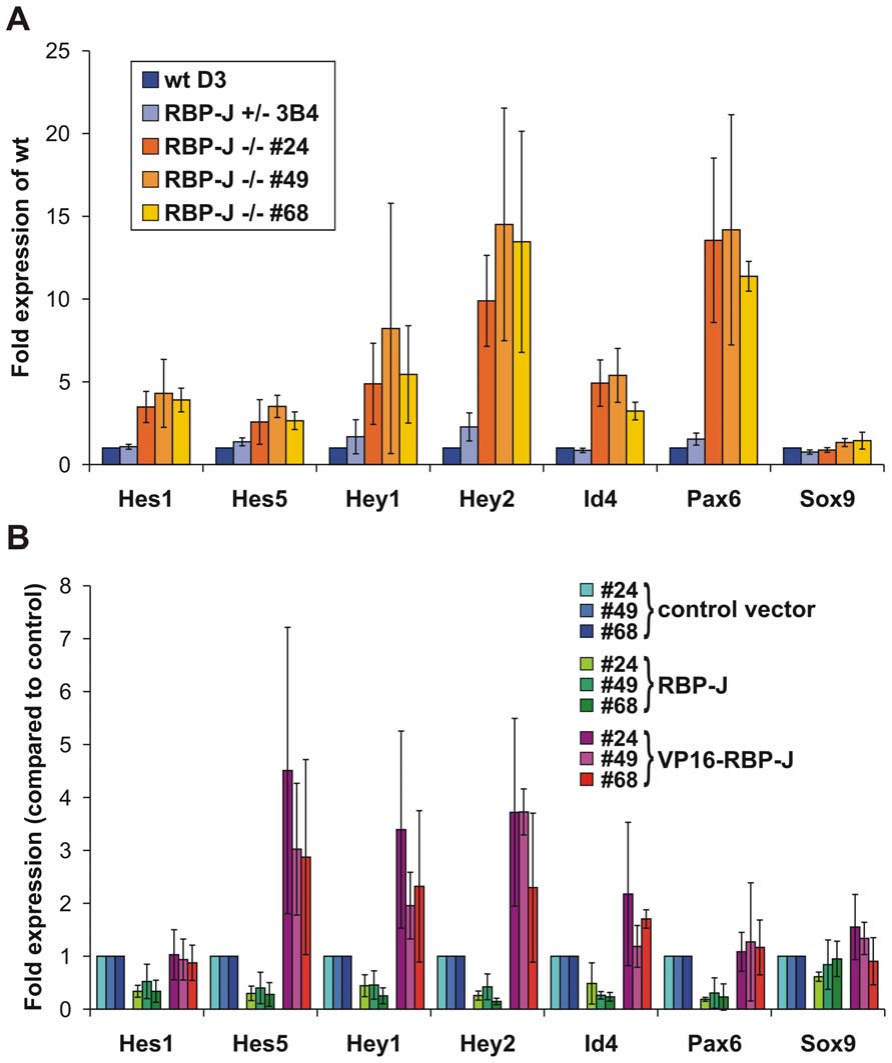
Expression of Notch1 target genes is altered in RBP-J deficient ES cells. mRNA levels were measured by qPCR in ES cells cultured in self renewal medium (ESCs). The error bars indicate the standard deviation from 3 independent experiments. (A) Expression of potentially direct Notch1 target genes is increased in RBP-J deficient ES cells. Fold expression in relation to the expression of RBP-J +/+ D3 cells is shown. (B) The increased expression of Notch1 target genes in RBP-J -/- ES cells is repressed by exogenous RBP-J expression and further increased by a transcriptionally active form of RBP-J (RBP-J-VP16). Fold expression in relation to the expression of cells transfected with the control vector is shown.

To confirm that the Notch1 target genes identified in our screen are regulated also under physiological conditions *in vivo* we used two approaches: First, we used genetic complementation of ESC that lack RBP-J [45], a transcription factor that plays a central role for canonical Notch signal transduction. To this end, expression levels of the target genes Hes1, Hes5, Hey1, Hey2, Id4, Pax6, and Sox9 that were likely regulated directly by activated Notch in ESC were analyzed in ESC clones that lacked RBP-J (RBP-J-/-) or had reduced expression of RBP-J (RBP-J+/-), as well as in RBP-J-/- ESC clones that were complemented with RBP-J (RBP-J rescue) or in RBP-J-/- ESC that expressed VP16-RBP-J, a transcriptionally active derivative of RBP-J, from a transgene (VP16-RBP-J rescue) [45]. As expected from the role of RBP-J as transcriptional repressor in the absence of Notch signaling, all three RBP-J-/- ESC clones analyzed expressed significantly higher amounts of Hes1, Hes5, Hey1, Hey2, Id4 and Pax6 (Fig. 11A). For all of these genes, re-expression of RBP-J from a transgene restored the repression (Fig. 11B). In line with active Notch signaling, expression levels for all of these genes were further increased in the presence of the transcriptionally active derivative of RBP-J (Fig. 11B), thus corroborating our array and qPCR data on Notch target genes. However, the regulation of Sox9 by Notch signaling appears to be more complex: Although activated Notch1 upregulated Sox9 expression, we did not observe significantly altered expression levels of Sox9 in ESC lacking RBP-J (Fig. 11A). One possibility for the unaltered expression levels of Sox9 in RBP-J-/- ESC could be that Sox9 is not suppressed by RBP-J, although it is regulated by Notch1 signaling. In the mammalian skin as well as during T helper cell differentiation, target repression by RBP-J does not play an important role [46], [47], suggesting that in these tissues Notch signals in an RBP-J independent manner. However, since the expression levels were influenced in some but not all RBP-J-/- ESC clones after re-expressing RBP-J or VP16-RBP-J from a transgene (Fig. 11B), a more likely explanation is that repression of Sox9 by RBP-J is dynamic and depends on other additional factors. Notably, in Drosophila, RBP-J occupancy on many Notch target promoters is a transient, dynamic process [48]. In both, *Drosophila* and mammalian cells, the presence of Notch^IC^ enhances RBP-J occupancy and irrespective of active repression by RBP-J or not, the binding of Notch^IC^ to RBP-J mediates the transcriptional switch to activate gene expresssion from target promoters. How RBP-J and Notch^IC^ binding to RBP-J-specific sequences at target gene promoters to activate gene expression is regulated in a cell-context dependent manner is an interesting issue for further investigation (see model below).

In our second approach we used knock-in reporter cell lines to confirm target gene activation by Notch1. Using ESC with a lacZ insertion in one myf5 allele [49] or ESC with a Venus gene, a modified version of yellow fluorescent protein, in the Runx1 locus [50], we generated stable ESC lines (Myf5-lacZ-NERT and Runx1-Venus-NERT), which express NERT, an OHT-inducible form of murine Notch1^IC^ [14], or control cell lines carrying the pCAG expression vector without the NERT cDNA (Myf5-LacZ and Runx1-Venus). Functionality of Notch1 pathway activation in Myf5-LacZ-NERT ESC and Runx1-Venus-NERT ESC was tested in undifferentiated ESC by analysis of Hes5 and HeyL expression after culturing the cells for 4 h in the presence or absence of OHT. All Myf5-LacZ-NERT ESC and Runx1-Venus-NERT ESC clones used for further study upregulated Hes5 and HeyL expression in the presence of OHT, while in control clones Hes5 and HeyL expression remained unchanged by the addition of OHT (data not shown). Using embryoid body culture, ESC were differentiated in the presence or absence of OHT to induce Notch signaling and analyzed at different times after plating. Venus expression was significantly induced by the addition of OHT in Runx1-Venus-NERT cells but not in Runx1-Venus control cells (Fig. 10B) at day 5 of mesodermal differentiation. Quantitative FACS analysis of mesodermal progenitor cells expressing Flk1 confirmed mesodermal differentiation and Notch activation, as previously described [14] (Fig. 10B). Similarly, activation of Notch signaling by the addition of OHT strongly induced the expression of β-Gal-positive cells in Myf5-lacZ-NERT cells but not in Myf5-LacZ control cells after 5 days of differentiation (Fig. 10C). Taken together, these results further confirm these genes to be regulated by activated Notch1.

### A mechanistic model for the role of Notch signaling in stem cell differentiation: Activated Notch promotes lineage entry by activating lineage specific transcription factors and expansion of lineage specific stem and progenitor cells by a negative feed back loop involving Hes/Hey and Id transcriptional repressors

**Figure 12:**
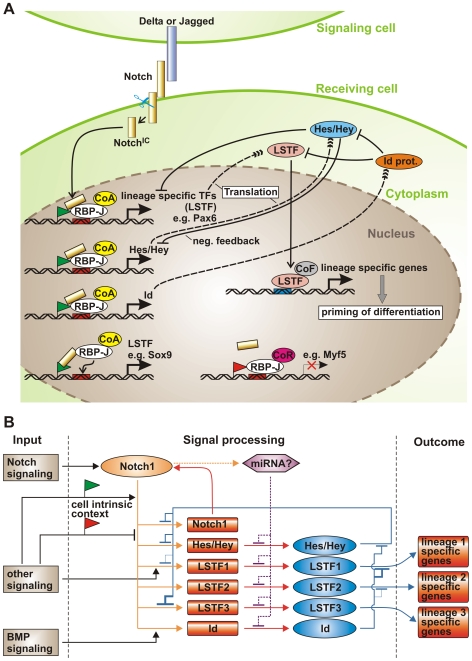
Model summarizing Notch1 cell-context dependent down-stream signaling. (A) Cell-based model. *CoR* co-repressor; *CoA* co-activator; *LSTF* lineage specific transcription factors; *green* and *red flag* activating or repressing chromatin marks. (B) Context-dependent regulatory circuit model. See text for details.

What is the consequence of Notch pathway activation in various cell types? Expression of constitutive active Notch promotes the generation of cell lineage specific stem cells and inhibits further differentiation [4]. We have shown here that activated Notch1 most likely directly induces lineage specific transcription factors (LSTFs), which are known to direct multipotent stem cells along a certain lineage and are required for differentiation, in a cell context dependent manner. We further found that Notch1 signaling at the same time also induced Notch target genes of the *Hes*/*Hey* family, which can block differentiation by negatively regulating expression and/or function of cell LSTFs, e.g. MyoD or Runx [51]. In addition we observed, that inhibiting protein synthesis augmented the transcriptional up-regulation of direct Notch target genes, indicating the presence of a negative feedback loop requiring the synthesis of a repressive protein. In this respect, the reported binding of Hes/Hey to RBP-J on their own promoters to inhibit their Notch-induced expression [52], suggests that Hes/Hey proteins play a critical role in this negative feedback loop. Taken together, we propose a model (Fig. 12) for the mechanistic function of Notch signaling in stem cell differentiation: Notch signaling activates the transcription of LSTF as well as Hes/Hey proteins. Specific chromatin marks or other transcription factors yet to be identified may determine cell context dependency for differential Notch^IC^ binding. The LSTFs then prime the cells for differentiation along the respective lineages, while the concomitant up-regulation of *Hes*/*Hey* genes counteracts further differentiation by interfering with expression or function of these LSTFs. Recently, a similar stimulation of the expression of a gene as well as the repressor of that gene by activated Notch was shown in *Drosophila* muscle progenitor cells [7]. Thus, activated Notch may promote lineage entry by activating LSTFs and expansion of lineage specific stem and progenitor cells by a negative feed back loop involving Hes/Hey transcriptional repressors.

Another layer of complexity as well as a link to other signaling pathways is provided by the differential regulation of Id proteins by activated Notch (Fig. 12A). During differentiation, Id proteins act as a short-lived brake withholding predetermined cells from differentiating, by associating with LSTFs and preventing them from binding DNA. At the same time, Id proteins can bind, possibly cell-context dependently, also to specific Hes proteins, thereby inhibiting the autorepression of certain *Hes* genes [53]. Thus, the cells are primed to adopt a 'pre-differentiation state' by the expression of LSTFs and held in check by opposing signals because they express inhibitors of the Id and Hes/Hey family at the same time. Further differentiation will then depend on the availability of LSTFs, which is regulated by the cell-context dependent amount and type of LSTFs, Id and Hes proteins (Fig. 12A). In addition to Notch signaling, the expression of Id proteins is positively regulated by TGFβ/BMP signaling in different tissues [41], contributing to the balance of transcription factor availability (Fig. 12B). Similarly, Hes and Hey proteins transduce and integrate signals from TGFβ/BMP, JAK-STAT, Ras and HIF signaling pathways [51]. On the other hand, LSTFs are also subject to regulation by several external signaling pathways, such as FGF, TGFβ/BMPs and Wnts (Fig. 12B). Furthermore, a positive feed-forward loop up-regulating Notch expression itself may ensure definitive determination into a specific lineage (Fig. 12B). Finally, work in *Drosophila*, zebrafish and *C. elegans* has demonstrated that miRNAs are direct transcriptional target genes of Notch signaling and regulate Notch target genes [54]–[56]. It is thus tempting to speculate, that miRNAs induced by activated Notch further influence the amount of certain transcription factors present and thus contribute to fine-tuning of the expression of Notch target genes also in mammals (Fig. 12B). In summary, we propose that Notch signaling ensures correct, coordinated and timed specification of stem cells by simultaneously activating LSTFs, differentiation-inhibitory proteins of the Hes/Hey and Id family, Notch receptors and miRNAs in an cell-intrinsic and cell-extrinsic context dependent manner.

## Materials and Methods

### Cell Culture

The culture of undifferentiated ESC was performed as described in Schroeder et al. [45]. To generate mesodermal cells, ESC were cultured in αMEM containing 15% pre-tested FCS (PAN, Germany), 100 µM 2-mercaptoethanol (differentiation medium) for 4 to 5 days [57] for transcriptome analysis. 5×10^4^ undifferentiated ESC per well were cultured in 6 well collagen type IV coated plates (Becton Dickinson, USA). Alternatively, for control of differentiation potential, 1×10^4^ undifferentiated ESC were cultured in 6 well plates containing confluent OP9 stromal cells [58]. The culture was then analyzed by flow cytometry for Flk-1+ cells. For the culture of cells in conditions favoring ectodermal or mesodermal differentiation, ESC grown on gelatine were washed and cultured for 4 h in the presence or absence of 1 µM OHT in either ES to ectoderm differentiation medium (GMEM, 10% Knockout Serum Replacement, 1% non essential amino acids, 1 mM sodium pyruvate (all Invitrogen), and 100 µM 2-mercaptoethanol) or in ES to mesoderm differentiation medium (Knockout-DMEM, 10% Knockout Serum Replacement (all Invitrogen), 1% pre-tested FCS (Cambrex), 1% non essential amino acids (Invitrogen), 2 mM L-Glutamine, and 100 µM 2-mercaptoethanol). To block protein synthesis, 50 µg/ml cycloheximide (CHX) was added for 4 h.

Runx1-Venus ESC [50] and Myf5-LacZ ESC [49] were stably transduced with 10 µg of pCAG-NERT^ΔOP^ vector or pCAG-IP control vector by electroporation and selected as described in Schroeder et al [45]. For in vitro differentiation of Myf5-LacZ-NERT ESC and Runx1-Venus-NERT ESC, ESC were differentiated using the hanging drop method as described [59]. Briefly, ESC were cultivated in hanging drops for 2 days until embryoid bodies were formed. Embryoid bodies were the transferred to bacteriological plates and further cultivated in suspension. Between day 5 and day 7 cells were plated on gelatine-coated tissue culture dishes and further incubated until analysis. ESC were electroporated and selected as previously described [14].

### Microarrays

RNAs were processed as described previously with minor changes [60] for use on Affymetrix GeneChips Mouse Genome 430 2.0 (Affymetrix, Santa Clara, CA, USA) and analyses were done with dChip Software. RNA from the different cell populations was isolated and treated with DNase I (RNeasy, Qiagen) and used for cDNA synthesis (5 µg total RNA, Expression Analysis Technical Manual, Affymetrix, Santa Clara, CA, USA). cRNA was generated (BioArray High-Yield Transcript Labeling kit, ENZO, Farmingdale, NY, USA) and hybridized to Affymetrix GeneChip Mouse Genome 430 2.0 Array (15 µg cRNA, 16 h, 45°C) that contain ∼45,000 probe sets of over 39,000 murine transcripts. GeneChip arrays were stained, washed and scanned according to the manufacturer's specifications. Principle component analysis (PCA) was performed using the R software (http://www.r-project.org/; [61]). Scanned GeneChip.DAT files were analyzed by dChip Software (Built date Aug 21, 2008; [62] (http://dchip.org). Microarrays were normalized to an array with median overall intensity (‘Control Mesoderm +OHT’) using the Invariant Set Normalization method [62]. Uninduced and OHT-induced samples of the same cell populations were compared. A gene was assigned as differentially regulated by Notch1, when it fulfilled the following criteria: (i) at least 2-fold up- or down-regulation, (ii) at least the higher expression value has to be judged as ‘present’ by the software, (iii) the absolute difference between induced/not-induced is higher than 50 (about 2-fold the mean background level of the ‘absent’ genes). For further analyses, redundant genes were removed as far as possible resulting in 465 genes and transcripts for all culture conditions applied. Data sets were deposited in NCBIs Gene Expression Omnibus (GEO, http://www.ncbi.nlm.nih.gov/geo/) and are accessible through GEO Series accession number GSE15268. Relative expression was visualized via heatmap using the MeV 4.2 of the TM4 software suite (http://www.tm4.org/; [63]). Values were Log_2_ transformed and normalized within each gene. Furthermore, genes were classified into Gene Ontology [64] or PANTHER groups [65] using the DAVID functional annotation analysis tool (http://david.abcc.ncifcrf.gov/). The GO and PANTHER terms used were: GOTERM_BP_ALL: GO:0006350∼transcription OR PANTHER_MF_ALL: MF00036: Transcription factor (‘Transcription and TFs’); GOTERM_BP_ALL: GO:0006915∼apoptosis; GOTERM_BP_ALL: GO:0030154∼cell differentiation; GOTERM_BP_ALL: GO:0007049∼cell cycle; GOTERM_BP_ALL: GO:0007154∼cell communication; GOTERM_BP_ALL: GO:0007155∼cell adhesion; GOTERM_BP_ALL: GO:0007242∼intracellular signaling cascade; GOTERM_BP_ALL: GO:0019222∼regulation of metabolic process; GOTERM_CC_ALL: GO:0005856∼cytoskeleton; GOTERM_BP_ALL: GO:0008283∼cell proliferation; PANTHER_BP_ALL: BP00248:Mesoderm development. Genes of the groups were reassigned to their induction values and visualized as a heatmap with the MeV 4.2 software.

### Real-time RT-PCR

Relative expression levels of 30 potential Notch1 target genes and Oct4 as a control were determined by real-time PCR on a 7900HT Fast Real-Time PCR System (Applied Biosystems) in 384-well PCR-plates (ABgene) using the TaqMan Gene Expression Assays-on-Demand system (Applied Bioystems, Foster City, USA) as described previously [14] with minor changes. Genes and assays used are itemized in Table S2. Relative expression levels of the genes were calculated in relation to Gapdh expression (Applied Biosystems) using the ΔCt method with efficiency values measured in a pilot experiment for each expression assay. Inductions by OHT were calculated with the ΔΔCt method [66] using Gapdh for normalization. Relative expression and inductions were visualized via heatmaps using MeV 4.2 software.

### Western Blots

60 µg of protein lysates from ESC or 5 d differentiated embryoid bodies were separated on a SDS-PAGE and blotted onto a nitrocellulose membrane. Immunoblots were carried out using antibodies against Sox9 (Santa Cruz Biotechnology, sc-17340), Pax6 (Covance, PRB-278P), Id4 (Santa Cruz Biotechnology, sc-491) and Actin (Santa Cruz Biotechnology, sc-1616R) in a 1∶200, 1∶1000, 1∶1000 and 1∶5000 dilution, respectively.

### LacZ Staining

Cells fixed with 2% formaldehyde were stained for β-Gal using standard procedures.

## Supporting Information

Table S1 Cell-context specific Notch1 target genes. List of cell-context specific Notch1 target genes identified by genome wide expression arrays in ESC under ectodermal and mesodermal differentiation conditions and in mesodermal progenitor cells.
(0.37 MB XLS)

Table S2 Gene expression assays of Notch1 target genes. Summary of gene expression assays used to determine relative expression levels of 30 potential Notch1 target genes after induction of Notch signaling.
(0.02 MB XLS)

Video S1 PCA shows that samples of each cell population, i.e. ESC (ESCe and ESCm) and mesodermal progenitor cells (Mesoderm), cluster together, regardless of the expression of the NERT protein or the presence of OHT.
(5.51 MB MOV)

## References

[1] Artavanis-Tsakonas S, Rand MD, Lake RJ (1999). Notch signaling: cell fate control and signal integration in development.. Science.

[2] Lai EC (2004). Notch signaling: control of cell communication and cell fate.. Development.

[3] Kopan R, Cagan R (1997). Notch on the cutting edge.. Trends Genet.

[4] Hurlbut GD, Kankel MW, Lake RJ, Artavanis-Tsakonas S (2007). Crossing path with Notch in the hyper-network.. Curr Opin Cell Biol.

[5] Iso T, Kedes L, Hamamori Y (2003). HES and HERP families: Multiple effectors of the Notch signaling pathway.. J Cell Physiol.

[6] Leimeister C, Externbrink A, Klamt B, Gessler M (1999). Hey genes: A novel subfamily of hairy- and enhancer of split related genes specifically expressed during mouse embryogenesis.. Mech Dev.

[7] Krejci A, Bernard F, Housden BE, Collins S, Bray SJ (2009). Direct response to Notch activation: Signaling crosstalk and incoherent logic.. Sci Signal.

[8] Poellinger L, Lendahl U (2008). Modulating Notch signaling by pathway-intrinsic and pathway-extrinsic mechanisms.. Curr Opin Genet Dev.

[9] Cormier S, Vandormael-Pournin S, Babinet C, Cohen-Tannoudji M (2004). Developmental expression of the Notch signaling pathway genes during mouse preimplantation development.. Gene Expr Patterns.

[10] Nemir M, Croquelois A, Pedrazzini T, Radtke F (2006). Induction of cardiogenesis in embryonic stem cells via downregulation of Notch1 signaling.. Circ Res.

[11] Swiatek PJ, Lindsell CE, del Amo FF, Weinmaster G, Gridley T (1994). Notch1 is essential for postimplantation development in mice.. Genes & Dev.

[12] Oka C, Nakano T, Wakeham A, de la Pompa JL, Mori C (1995). Disruption of the mouse RBP-J kappa gene results in early embryonic death.. Development.

[13] Conlon RA, Reaume AG, Rossant J (1995). Notch1 is required for the coordinate segmentation of somites.. Development.

[14] Schroeder T, Meier-Stiegen F, Schwanbeck R, Eilken H, Nishikawa S (2006). Activated Notch1 alters differentiation of embryonic stem cells into mesodermal cell lineages at multiple stages of development.. Mech Dev.

[15] Lowell S, Benchoua A, Heavey B, Smith A (2006). Notch promotes neural lineage entry by pluripotent embryonic stem cells.. PLoS Biology.

[16] Schroeder T, Kohlhof H, Rieber N, Just U (2003). Notch signaling induces multilineage myeloid differentiation and up-regulates PU.1 expression.. J Immunol.

[17] Schwanbeck R, Schroeder T, Henning K, Kohlhof H, Rieber N (2008). Notch signaling in embryonic and adult myelopoiesis.. Cells Tissues Organs.

[18] Henning K, Schroeder T, Schwanbeck R, Rieber N, Bresnick EH (2007). mNotch1 signaling and erythropoietin cooperate in erythroid differentiation of multipotent progenitor cells and upregulate beta-globin.. Exp Hematol.

[19] Main H, Lee KL, Yang H, Haapa-Paananen S, Edgren H (2010). Interactions between Notch- and hypoxia-induced transcriptomes in embryonic stem cells.. Exp Cell Res.

[20] Taylor MK, Yeager K, Morrison SJ (2007). Physiological Notch signaling promotes gliogenesis in the developing perpheral and central nervous system.. Development.

[21] Muto A, Iida A, Satoh S, Watanabe S (2009). The group E Sox genes Sox8 and Sox9 are regulated by Notch signaling and are required for Müller glial cell development in mouse retina.. Exp Eye Res.

[22] Zong Y, Panikkar A, Xu JF, Antoniou A, Raynaud P (2009). Notch signaling controls liver development by regulating biliary differentiation.. Development.

[23] Osumi N, Shinohara H, Numayama-Tsuruta K, Maekawa M (2008). Pax6 transcription factor contributes to both embryonic and adult neurogenesis as a multifunctional regulator.. Stem Cells.

[24] Suter DM, Tirefort D, Julien S, Krause KH (2009). A Sox1 to Pax6 switch drives neuroectoderm to radial glia progression during differentiation of mouse embryonic stem cells.. Stem Cells.

[25] Onuma Y, Takahashi S, Asashima M, Kurata S, Gehring WJ (2002). Conservation of Pax 6 function and upstream activation by Notch signaling in eye development of frogs and flies.. Proc Natl Acad Sci U S A.

[26] Levanon D, Groner Y (2004). Structure and regulated expression of mammalian RUNX genes.. Oncogene.

[27] Kurokawa M (2006). AML1/Runx1 as a versatile regulator of hematopoiesis: Regulation of its function and a role in adult hematopoiesis.. Int J Hematol.

[28] Lebestky T, Jung SH, Banerjee U (2003). A Serrate-expressing signaling center controls Drosophila hematopoiesis.. Genes & Dev.

[29] Nakagawa M, Ichikawa M, Kumano K, Goyama S, Kawazu M (2006). AML1/Runx1 rescues Notch1-null mutation-induced deficiency of para-aortic splanchnopleural hematopoiesis.. Blood.

[30] Burns CE, Traver D, Mayhall E, Shepard JL, Zon LI (2005). Hematopoietic stem cell fate is established by the Notch-Runx pathway.. Genes & Dev.

[31] Vasyutina E, Lenhard D, Birchmeier C (2007). Notch function in myogenesis.. Cell Cycle.

[32] Arnold HH, Braun T (2000). Genetics of muscle determination and development.. Curr Top Dev Biol.

[33] Vasyutina E, Lenhard D, Wende H, Erdmann E, Epstein J (2007). RBP-J (Rbpsuh) is essential to maintain muscle progenitor cells and to generate satellite cells.. Proc Natl Acad Sci U S A.

[34] Schuster-Gossler K, Cordes R, Gossler A (2007). Premature myogenic differentiation and depletion of progenitor cells cause severe muscle hypotrophy in Delta1 mutants.. Proc Natl Acad Sci U S A.

[35] Kuroda K, Tani S, Tamura K, Minoguchi S, Kurooka H (1999). Delta-induced Notch signaling mediated by RBP-J inhibits MyoD expression and myogenesis.. J Biol Chem.

[36] Asakura A, Hirai H, Kablar B, Morita S, Ishibashi J (2007). Increased survival of muscle stem cells lacking the MyoD gene after transplantation into regenerating skeletal muscle.. Proc Natl Acad Sci USA.

[37] Gensch N, Borchardt T, Schneider A, Riethmacher D, Braun T (2008). Different autonomous myogenic cell populations revealed by ablation of Myf5-expressing cells during mouse embryogenesis.. Development.

[38] Haldar M, Karan G, Tvrdik P, Capecchi MR (2008). Two cell lineages, myf5 and myf5-independent, participate in mouse skeletal myogenesis.. Dev Cell.

[39] Ustanina S, Carvajal J, Rigby P, Braun T (2007). The myogenic factor Myf5 supports efficient skeletal muscle regeneration by enabling transient myoblast amplification.. Stem Cells.

[40] Buas M, Kabak S, Kadesh T (2009). Inhibition of myogenesis by Notch: Evidence for multiple pathways.. J Cell Physiol.

[41] Ruzinova MB, Benezra R (2003). Id proteins in development, cell cycle and cancer.. Trends Cell Biol.

[42] Baonza A, de Celis JF, Garcia-Bellido A (2000). Relationships between extramacrochaetae and Notch signalling in Drosophila wing development.. Development.

[43] Reynaud-Deonauth S, Zhang H, Afouda A, Taillefert S, Beatus P (2002). Notch signaling is involved in the regulation of Id3 gene transcription during Xenopus embryogenesis.. Differentiation.

[44] Yokota Y (2001). Id and development.. Oncogene.

[45] Schroeder T, Fraser ST, Ogawa M, Nishikawa S, Oka C (2003). Recombination signal sequence-binding protein Jkappa alters mesodermal cell fate decisions by suppressing cardiomyogenesis.. Proc Natl Acad Sci U S A.

[46] Ong CT, Sedy JR, Murphy KM, Kopan R (2008). Notch and presenilin regulate cellular expansion and cytokine secretion but cannot instruct Th1/Th2 fate acquisition.. PLoS ONE.

[47] Demehri S, Liu Z, Lee J, Lin MH, Crosby SD (2008). Notch-deficient skin induces a lethal systemic B-lymphoproliferative disorder by secreting TSLP, a sentinel for epidermal integrity.. PLoS Biol.

[48] Krejci A, Bray S (2007). Notch activation stimulates transient and selecive binding of Su(H)/CSL to target enhancers.. Genes & Dev.

[49] Braun T, Arnold HH (1996). myf-5 and myoD genes are activated in distinct mesenchymal stem cells and determine different skeletal muscle cell lineages.. EMBO J.

[50] Hirai H, Samokhvalov IM, Fujimoto T, Nishikawa S, Imanishi J (2005). Involvement of Runx1 in the down-regulation of fetal liver kinase-1 expression during transition of endothelial cells to hematopoietic cells.. Blood.

[51] Fischer A, Gessler M (2007). Delta-Notch-and then? Protein interactions and proposed modes of repression by Hes and Hey bHLH factors.. Nucleic Acids Research.

[52] King IN, Kathiriya IS, Murakami M, Nakagawa M, Gardner KA (2006). Hrt and Hes negatively regulate Notch signaling through interaction with RBP-Jkappa.. Biochem Biophys Res Commun.

[53] Bai G, Sheng N, Xie Z, Bian W, Yokota Y (2007). Id sustains Hes1 expression to inhibit precocious neurogenesis by releasing negative autoregulation of Hes1.. Dev Cell.

[54] Yoo AS, Greenwald I (2005). LIN-12/Notch activation leads to microRNA-mediated down-regulation of Vav in C. elegans.. Science.

[55] Lai EC, Tam B, Rubin GM (2005). Pervasive regulation of Drosophila Notch target genes by GY-box-, Brd-box-, and K-box-class microRNAs.. Genes & Dev.

[56] Thatcher EJ, Flynt AS, Li N, Patton JR, Patton JG (2007). miRNA expression analysis during normal zebrafish development and following inhibition of the hedgehog and Notch signaling pathways.. Dev Dynamics.

[57] Hirashima M, Kataoka H, Nishikawa S, Matsuyoshi N (1999). Maturation of embryonic stem cells into endothelial cells in an in vitro model of vasculogenesis.. Blood.

[58] Kodama H, Nose M, Niida S, Nishikawa S (1994). Involvement of the c-kit receptor in the adhesion of hematopoietic stem cells to stromal cells.. Exp Hematol.

[59] Wobus AM, Guan K, Yang HT, Boheler KR (2002). Embryonic stem cells as a model to study cardiac, skeletal muscle, and vascular smooth muscle cell differentiation.. Methods Mol Biol.

[60] Ruau D, Ju XS, Zenke M (2006). Genomics of TGF-beta1 signaling in stem cell commitment and dendritic cell development.. Cell Immunol.

[61] Ihaka R, Gentleman R (1996). A language for data analysis and graphics.. J Comput Graph Stat.

[62] Li C, Wong WH (2001). Model-based analysis of oligonucleotide arrays: Expression index computation and outlier detection.. Proc Natl Acad Sci U S A.

[63] Saeed AI, Sharov V, White J, Li J, Liang W (2003). TM4: a free, open-source system for microarray data management and analysis.. Biotechniques.

[64] Ashburner M, Ball CA, Blake JA, Botstein D, Butler H (2000). Gene ontology: tool for the unification of biology. The Gene Ontology Consortium.. Nat Genet.

[65] Thomas PD, Campbell MJ, Kejariwal A, Mi H, Karlak B (2003). PANTHER: a library of protein families and subfamilies indexed by function.. Genome Res.

[66] Livak J, Schmittgen T (2001). Analysis of relative gene expression data using real-time quantitative PCR and the 2(-Delta Delta C(T)) Method.. Methods.

[67] Schug J (2008). Using TESS to predict transcription factor binding sites in DNA sequence.. Curr Protoc Bioinformatics Chapter.

[68] Ong CT, Cheng HT, Chang LW, Ohtsuka T, Kageyama R (2006). Target selectivity of vertebrate notch proteins. Collaboration between discrete domains and CSL-binding site architecture determines activation probability.. J Biol Chem.

